# How to deal with the high condition number of the noise covariance matrix of gravity field functionals synthesised from a satellite-only global gravity field model?

**DOI:** 10.1007/s00190-018-1136-0

**Published:** 2018-03-23

**Authors:** R. Klees, D. C. Slobbe, H. H. Farahani

**Affiliations:** Delft University of Technology, Stevinweg 1, 2628 CN Delft, The Netherlands

**Keywords:** Local quasi-geoid, Least-squares, Global gravity field model, Noise covariance matrix, Regularisation, Unified theory of least-squares, Spherical radial basis functions

## Abstract

The posed question arises for instance in regional gravity field modelling using weighted least-squares techniques if the gravity field functionals are synthesised from the spherical harmonic coefficients of a satellite-only global gravity model (GGM), and are used as one of the noisy datasets. The associated noise covariance matrix, appeared to be extremely ill-conditioned with a singular value spectrum that decayed gradually to zero without any noticeable gap. We analysed three methods to deal with the ill-conditioned noise covariance matrix: Tihonov regularisation of the noise covariance matrix in combination with the standard formula for the weighted least-squares estimator, a formula of the weighted least-squares estimator, which does not involve the inverse noise covariance matrix, and an estimator based on Rao’s unified theory of least-squares. Our analysis was based on a numerical experiment involving a set of height anomalies synthesised from the GGM GOCO05s, which is provided with a full noise covariance matrix. We showed that the three estimators perform similar, provided that the two regularisation parameters each method knows were chosen properly. As standard regularisation parameter choice rules do not apply here, we suggested a new parameter choice rule, and demonstrated its performance. Using this rule, we found that the differences between the three least-squares estimates were within noise. For the standard formulation of the weighted least-squares estimator with regularised noise covariance matrix, this required an exceptionally strong regularisation, much larger than one expected from the condition number of the noise covariance matrix. The preferred method is the inversion-free formulation of the weighted least-squares estimator, because of its simplicity with respect to the choice of the two regularisation parameters.

## Introduction

In local gravity field modelling, a global gravity field model (GGM) may be considered as another noisy dataset next to the local datasets such as terrestrial and shipboard gravity anomalies, airborne gravity disturbances, radar altimeter deflections of the vertical or along-track height anomaly differences. In Stokes-based approaches, part of the information in the form of noise degree variances is routinely used for the modification of the Stokes kernel (e.g. Sjöberg 1980, 1981; Wenzel 1981). Sjöberg (2005, 2011) extended the formalism, which now allows to include a full noise covariance matrix of the spherical harmonic coefficients.

So far, no publication is known to the authors, which indeed used a full noise covariance matrix of the GGM in local gravity field modelling. The only exception is Klees et al. (2017). This may be explained among others by the fact that in the past, a full noise covariance matrix was not available or was considered as being not reliable enough. This has changed recently with the latest generation of GGMs, which are mainly based on low-low satellite-to-satellite (ll-SST) tracking data of the Gravity Recovery and Climate Experiment (GRACE) mission, satellite gravity gradiometry (SGG) data of the Gravity field and steady-state Ocean Circulation Explorer (GOCE) mission, and high-low satellite-to-satellite (hl-SST) tracking data of GRACE, GOCE and many other low-earth orbiters. Post-fit residual analysis (e.g. Farahani et al. 2013), has become a powerful tool to improve the noise model of the satellite data. Numerically efficient algorithms were developed, which propagate the full data noise covariance matrices into the estimated spherical harmonic coefficients of the GGM. One example is GOCO05s (Mayer-Gürr et al. 2015), which is complete to degree 280 and provided with a full noise covariance matrix. This matrix has been propagated using the law of covariance propagation from the noise covariance matrices of the individual datasets used to compute GOCO05s. The noise covariance matrices of the individual datasets are based on a post-fit residual analysis and modelled using empirical covariance functions and ARMA models, respectively, depending on the dataset.

When estimating a local model of the disturbing potential using least-squares techniques, there are basically two possibilities to include ll-SST, hl-SST, and SGG data: i) using these data directly as observations (e.g. Eicker 2008; Eicker et al. 2014; Bucha et al. 2015; Naeimi and Bouman 2017) or ii) using the spherical harmonic coefficients of the GGM (e.g. Schmidt et al. 2007; Klees et al. 2017). In the former case, the noise covariance matrices of the satellite datasets are well-conditioned. However, dealing with original data may increase the numerical complexity of the parameter estimation significantly, in particular when using ll-SST data of the GRACE satellite gravity mission with a proper noise model. However, it also comes at a price. The spherical harmonic coefficients cannot be used directly as observations in local gravity field modelling (Klees et al. 2017). Instead, gravity field functionals (e.g. disturbing potential values, height anomalies, or gravity disturbances) need to be synthesised from the spherical harmonic coefficients at the Earth’s surface or at altitude. The associated noise covariance matrix has to be computed from the noise covariance matrix of the spherical harmonic coefficients using the law of covariance propagation.

Propagating the noise covariance matrix of spherical harmonic coefficients, into a set of gravity field functionals over a local area at the Earth’s surface or at altitude provides a noise covariance matrix that has a gradually decreasing singular value spectrum without any noticeable gap (cf. Sect. 2). Depending on the point density, the noise covariance matrix may be extremely ill-conditioned, meaning that the spectral norm condition number is much larger than $\varepsilon ^{-1}$, where $\varepsilon$ is the relative rounding error unit of IEEE 754 double precision arithmetic. In this study, we investigate three approaches to deal with the ill-conditioned noise covariance matrix: (i) applying Tikhonov regularisation (Tikhonov 1963) to the noise covariance matrix in combination with the standard formula for the weighted least-squares estimator; (ii) using an alternative formula for the weighted least-squares estimator, which does not require to invert the noise covariance matrix (Grafarend and Schaffrin 1993); and (iii) using an estimator based on the theory of unified least-squares (Rao 1971, 1973, 1978), which was designed among others to deal with a rank-deficient noise covariance matrix.

The reminder of the paper is organised as follows: in Sect. 2, we investigate several parameters, which have an influence on the condition number of the noise covariance matrix of gravity field functionals when propagated from a full noise covariance matrix of spherical harmonic coefficients of a state-of-the-art GGM. It appears that the condition number of the noise covariance matrix increases exponentially with the density of the points at which the gravity field functionals are synthesised. In Sect. 3, we investigate the minimum point density, which is required to reduce the functional model error below the noise level in the data. In Sect. 4, we introduce the three methods investigated in this study. Aspects such as the experimental setup, and the choice of various regularisation parameters each method requires to be made are the subject of Sect. 5. In Sect. 6, we present and discuss the results of the numerical experiments. Section 7 provides a summary and the conclusions.

## The condition number of the noise covariance matrix of a gravity field functional synthesised from a satellite-only GGM

When propagating the full noise covariance matrix of a spherical harmonic model of the Earth’s gravity field into gravity field functionals using the law of covariance propagation, the condition number of the gravity field functionals’ noise covariance matrix essentially depends on a number of parameters, among others, the density of the data points, the size of the data area, the maximum degree of the GGM, the altitude of the data points, and the type of gravity field functional.

### Impact of the point density

**Fig. 1:**
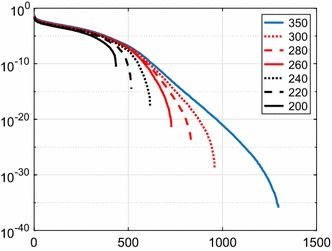
Singular values in units of square metres of noise covariance matrices of height anomalies on Reuter grids of different densities (cf. Table 1 for details about the grids). The insert shows the Reuter grid control parameter *N*, which controls the point density. A grid parameter of *N* provides the minimum number of data points to represent a spherical harmonic model of the disturbing potential complete to degree $N-1$ (cf. Freeden et al. 1998). The noise covariance matrix was computed using covariance propagation from the full noise covariance matrix of the unregularised GOCO05s spherical harmonic model complete to degree 200. All computations were done with 38 decimal digits using the Advanpix Multiprecision Toolbox for MATLAB, to avoid that small singular values are computed with large relative errors

Figure 1 depicts the singular values of noise covariance matrices of height anomalies, which were synthesised at the nodes of Reuter grids of varying density. The Reuter grid (Reuter 1982) is one of the point distributions frequently used in SRBF modelling (e.g. Eicker 2008). The grid width along the meridians is constant. Along the parallels, the number of grid points decreases with increasing latitude to achieve an equidistant distribution on the sphere. The Reuter grid knows one control parameter, denoted *N*, which determines the distance between the grid nodes, i.e. the point density. The number of grid nodes is close to but does not exceed $2 + {4 \over \pi } N^2$ over the whole surface of the sphere (Reuter 1982; Freeden et al. 1998). The grids were located on the Earth’s surface and covered an area bounded by $44^\circ$–$68^\circ$N and $11^\circ$W–$15^\circ$E. Each height anomaly noise covariance matrix was computed by covariance propagation from the full noise covariance matrix of the unregularised GOCO05s spherical harmonic model complete to degree $L=200$. Truncating GOCO05s at degree 200 makes sense here as beyond that degree the commission error grows exponentially and may quickly attain values much larger than the uncertainty of local datasets (e.g. terrestrial gravity anomalies) used in local gravity field modelling. For instance, over the data area defined above, we found that the average height anomaly noise standard deviation from the unregularised GOCO05s GGM complete to degree 280 can be modelled as $e^{0.03676 L - 6.5191}$ m, which is 2.3 cm at $L=200$, but already about 15 cm at $L=250$ and more than 40 cm at $L=280$. This exponential grow of the commission error implies that in local gravity field modelling, it does not make sense to use GOCO05s up to the maximum degree, except some areas in the world where terrestrial gravity datasets have an even poorer quality.

**Table 1:** Characteristic numbers of the Reuter grids used in Fig 1 and the condition number of the noise covariance matrix of height anomalies synthesised on these grids from the GOCO05s noise covariance matrix of spherical harmonic coefficients complete to degree 200

*N*	# Points	Mean point distance	Condition number
200	435	0d54m00s	$9.7 \cdot 10^8$
220	516	0d49m05s	$6.3 \cdot 10^{12}$
240	617	0d45m00s	$1.4 \cdot 10^{16}$
260	729	0d41m32s	$6.6 \cdot 10^{19}$
280	834	0d38m34s	$7.0 \cdot 10^{22}$
300	958	0d36m00s	$1.9 \cdot 10^{27}$
350	1297	0d30m51s	$4.0 \cdot 10^{34}$

The grid was located on the Earth’s surface and covered an area bounded by $44^\circ$–$68^\circ$N and $11^\circ$W–$15^\circ$E. The condition numbers were computed with 38 decimal digits using the Advanpix Multiprecision Toolbox for MATLAB, to avoid that small singular values are computed with large relative errors

Figure 1 and Table 1 reveal that the condition number increases exponentially with increasing point density. It may easily exceed $10^{15}$, which implies the loss of all significant digits in IEEE 754 double precision arithmetic when computing its inverse.

The point density needs to be chosen with care. The results of Table 1 suggest that a low point density is to be preferred. On the other hand, the point density must be high enough (i) to preserve all information contained in the GGM over the area of interest, and (ii) to guarantee that the error of the local model of the disturbing potential is negligible compared to the effect of the data noise. Therefore, in Sect. 3, we will determine the minimum point density needed to reconstruct a GGM over a local area of interest with a model error significantly below the effect of the data noise.

### Impact of the size of the data area

We also found that the condition number of the data noise covariance matrix depends on the size of the data area. Over the range of data areas we investigated (up to a size of $34^\circ \times 36^\circ$), we found that the larger the data area, the larger the condition number. For instance, if the height anomalies were located on a Reuter grid with $N=240$, the condition number of the height anomaly noise covariance matrix was $4.0\cdot 10^{10}$ for a data area of $49^\circ {-}63^\circ$N, $6^\circ$W–$10^\circ$E, and increased to $1.4 \cdot 10^{16}$ and $1.9 \cdot 10^{22}$ for a data area of $44^\circ {-}68^\circ$N, $11^\circ$W–$15^\circ$E and $39^\circ {-}73^\circ$N, $16^\circ$W–$20^\circ$E, respectively. Similar condition numbers were obtained when other areas on the globe were chosen. On the other hand, it is well known that if the data area is not global, the disturbing potential is distorted with the largest distortions along the border of the data area. These distortions, which are usually referred to as edge effects, reduce towards the centre of the data area (e.g. Schachtschneider et al. 2010). Hence, to reduce the distortions, the data area has to be chosen larger than the area of interest. For instance, Naeimi (2013) suggested an extension of the data area beyond the area of interest by ${10{,}000 \over L_{\tiny {\text{ min }}}}$ km assuming that the data have no energy at spherical harmonic degrees $\le L_{\tiny {\hbox {min}}}$. In our study, we found empirically that an extension of $5^\circ$ reduces the distortions over the area of interest to a level below 2 mm. This result is independent of the size of the area of interest as shown in (Schachtschneider et al. 2010). Hence, only for data areas much smaller than the one considered in the numerical experiments of Sect. 5, the condition number of the noise covariance matrix may be small enough to allow for a stable computation of the inverse.

### Impact of other parameters

Other parameters which may influence the condition number of the data noise covariance matrix, comprise the maximum degree of the GGM, the type of gravity field functional, and the altitude of the data grid.

The dependency on the maximum degree of the GGM was found to be moderate. To understand this result, we must remember that when increasing the maximum degree of the GGM, we also have to use a denser dataset. For instance, when we used GOCO05s complete to degree 200 and located the data points on a Reuter grid with $N=201$, the condition number was $9.7 \cdot 10^{8}$. When we used GOCO05s complete to degree 280 and located the data on a Reuter grid with $N=281$, the condition number increased to $2.6 \cdot 10^{10}$. We obtained similar results when using larger values of *N*.

The effect of the type of gravity field functionals and the altitude of the data grid on the condition number of the data noise covariance matrix appeared to be marginal. For instance, when we used gravity disturbances instead of height anomalies, the condition number increased by a factor of only 4.6; when we used data at an altitude of 250 km instead of data on the Earth’s surface, the condition number increased by a factor of only 1.6.

Supported by these results, we will represent the GGM in terms of height anomalies at points located on the Earth’s surface, and will use a data area which is $5^\circ$ larger than the area of interest in Sects. 3, 5, and 6.

## Model error as function of the data point density

To investigate the model error as function of the data point density, we first needed to select a local model of the disturbing potential. Without loss of generality, we used a spherical radial basis function (SRBF) model. SRBFs have been used successfully in many studies of local gravity field and (quasi-) geoid modelling (e.g. Klees et al. 2008; Eicker 2008; Wittwer 2009; Bentel et al 2013; Naeimi 2013; Slobbe 2013; Lin et al. 2014; Bentel and Schmidt 2016; Lieb et al. 2016; Bucha et al. 2016; Naeimi and Bouman 2017).

The following experimental setup was chosen. The disturbing potential was set equal to the regularised GOCO05s spherical harmonic model from degree 151–200. The area of interest was bounded by $49^\circ$–$63^\circ$N and $6^\circ$W–$10^\circ$E (i.e. the size is $1500 \times 1000$ km). Noise-free height anomalies were generated on a Reuter grid with control parameter *N* located on the Earth’s surface. The latter was represented by a smoothed version of the General Bathymetric Chart of the Oceans 2008 (GEBCO_08) grid, a terrain model for ocean and land with a spatial resolution of $30''$ (www.gebco.net). The local model of the disturbing potential comprised Poisson wavelets of order 3 (Holschneider et al. 2003), which were truncated at the maximum degree 200 of the disturbing potential. The poles of the Poisson wavelets were located at a constant depth beneath the data points. Though working with truncated Poisson wavelets is not necessary for the experiments of Sect. 5, it is a prerequisite when combining the GGM dataset with high-resolution local datasets as shown in (Klees et al. 2017). It ensures spectral consistency between the GGM dataset and its noise covariance matrix and the SRBF model of the disturbing potential.

We selected a number of Reuter grids with different control parameters ranging from $N=201$ to $N=350$. For each grid, we generated noise-free height anomalies and estimated the SRBF model parameters using ordinary least-squares. We always applied Tikhonov regularisation with a unit regularisation matrix. The estimated SRBF model of the disturbing potential was used to synthesise height anomalies on an equal-angular control grid of width $27'00''$ covering the area of interest, and comprising 1085 grid points. The differences between them and the height anomalies directly synthesised from the spherical harmonic coefficients of the disturbing potential are referred to as “model errors”.

Table 2 shows the statistics of the model errors for various choices of the Reuter grid control parameter *N*. For each *N*, the statistics refer to a least-squares solution obtained for a depth of the Poisson wavelets and a regularisation parameter providing the smallest model error among a set of candidate depths and regularisation parameters. As expected, the model error decreases with increasing point density. What model error is acceptable depends on the impact of data noise on the estimated quasi-geoid model. An indication of the latter is obtained when propagating the GOCO05s noise covariance matrix into height anomalies. For GOCO05s complete to degree 200, the height anomaly noise standard deviations range from 1.6  to 2.7 cm over the area of interest. The maximum absolute model error should be significantly smaller than 1.6 cm. Table 2 shows that the choice $N = 240$ provides a maximum absolute model error of 2 mm, i.e. a factor of 8 below the smallest height anomaly noise standard deviation. Therefore, we used $N=240$ in the numerical experiments of Sect. 5.

**Table 2:** SRBF model error in terms of height anomalies (in units of cm) as a function of the Reuter grid control parameter *N*

*N*	# Points	Distance (km)	Min	Max	Mean	RMS
201	435	99	$-\,6.66$	7.24	$9.6 \cdot 10^{-3}$	1.77
210	470	95	$-\,3.28$	2.93	$3.5 \cdot 10^{-2}$	0.87
220	516	91	$-\,0.84$	0.68	$7.82 \cdot 10^{-3}$	0.20
240	617	83	$-\,0.20$	0.19	$-\,6.4 \cdot 10^{-4}$	0.04
260	729	77	$-\,0.05$	0.05	$-\,3.0 \cdot 10^{-4}$	0.01
280	834	72	$-\,0.03$	0.03	$-\,1.7 \cdot 10^{-4}$	0.01
300	958	67	$-\,0.03$	0.03	$-\,2.0 \cdot 10^{-6}$	0.01
350	1297	57	$-\,0.02$	0.02	$-\,4.8 \cdot 10^{-6}$	0.01

The condition number of the height anomaly noise covariance matrix for data on a Reuter grid with $N=240$ is $1.4 \cdot 10^{16}$ (cf. Table 1). A straightforward inversion of this matrix would imply the loss of all significant digits in IEEE 754 double precision arithmetic. Reducing the SRBF model error further would require an even denser grid of height anomalies, which further increases the condition number according to Fig. 1 and Table 1. For instance, using a Reuter grid with $N=350$ reduces the model error to 0.2 mm, but increases the condition number of the height anomaly noise covariance matrix $4.0 \cdot 10^{34}$. How to deal with extremely ill-conditioned noise covariance matrices of height anomalies is the subject of Sect. 4.

Note that the model error depends on various settings such as the type of the SRBF, the grid used to locate the poles of the SRBFs and the data points, respectively, the area of interest, the extension of the parameterisation area beyond the data area, etc. Hence, each choice may lead to a different model error as function of the data point density.

In our experiments, the parameterisation area was chosen identical to the data area. Several studies suggest to extend the parameterisation area beyond the data area (e.g. Naeimi 2013; Bentel et al. 2013a; Eicker et al. 2014; Bucha et al. 2016). This raises the question whether in this case the model error can be made small enough to avoid any oversampling at the benefit of a condition number small enough to allow for a direct inversion without regularisation. In “Appendix A”, we present the results of a series of experiments designed to investigate this question. They reveal that when extending the parameterisation area beyond the data area, the model error statistics improve, though we still need to oversample by at least a factor of 1.2 to obtain a maximum absolute model error which is comparable to the one obtained without an extension of the parameterisation area.

Next to the experiments presented in “Appendix A”, we did a series of experiments with different types of SRBFs [Shannon kernel (Freeden et al. 1998) and point mass kernel (Hardy and Göpfert 1975)] and different point distributions to locate the poles of the SRBFs and the data points, respectively [Reuter grid, Fibonacci grid (Gonzalez 2010), triangle vertex grid (Eicker 2008)]. For each chosen setup, we could find parameter settings, which provide model error statistics as function of the data point density similar to the ones shown in Table 2. Importantly, we always needed to oversample by at least a factor of 1.2 to reduce the model errors to a level below the effect of data noise by at least a factor of 5. Moreover, the condition numbers of the corresponding data noise covariance matrices were identical to within a factor of 5.

## Dealing with the ill-conditioned noise covariance matrix

In this study, we investigate three approaches to deal with the high condition number of the noise covariance matrix: (i) apply Tikhonov regularisation (Tikhonov 1963) to the ill-conditioned data noise covariance matrix and use the standard formula for the weighted least-squares estimator, (ii) use a formula of the weighted least-squares estimator, which does not require the computation of the inverse of the noise covariance matrix (Grafarend and Schaffrin 1993), and (iii) use Rao’s generalised least-squares estimator (Rao 1971, 1973, 1978).

The functional model of the GGM dataset is written as a linear Gauss–Markov model,

1 $$\begin{aligned} E\{\mathbf d\} = \mathbf A\,\mathbf c,\;\; D\{\mathbf d\} = \mathbf {C} = \sigma ^2 \mathbf {Q}, \end{aligned}$$

where *E* is the expectation operator and *D* is the dispersion operator, $\mathbf d$ is the vector of height anomalies, $\mathbf c$ is the vector of SRBF coefficients, $\mathbf C$ is the height anomaly noise covariance matrix, $\mathbf Q$ is the cofactor matrix, and $\sigma ^2$ is the variance factor. An element $A_{ji}$ of the design matrix is equal to $\varPsi (x_j,z_i)$, where $\varPsi$ is the SRBF, $z_i$ is the coordinate vector of the i-th SRBF pole, and $x_j$ is the coordinate vectors of the j-th data point, i.e.

2 $$\begin{aligned} A_{ji}= & {} \varPsi (x_j,z_i) = {R \over |x_j|} \sum _{l=0}^L \lambda _l \Big ( {|z_i| \over |x_j|}\Big )^l\, Q_l(\hat{x}_j \cdot \hat{z}_i), \nonumber \\&\quad x \in \overline{\hbox {ext} \sigma _R}, \; z_i \in \hbox {int} \sigma _R. \end{aligned}$$

*L* is the degree up to which the GGM is used when synthesising the data, $\{\lambda _l: l=1 \ldots L\}$ are the Legendre coefficients of the SRBF with respect to the surface $\sigma _R$ of a sphere of radius *R*, $\hat{x}_j = {x_j \over |x_j|}$ and $\hat{z}_i = {z_i \over |z_i|}$ are points on the unit sphere, and $Q_l$ is the reproducing kernel of the space of spherical harmonics of degree *l*. For the Poisson wavelets of order 3, it is $\lambda _l = l^3$.

### Regularisation of the noise covariance matrix

Suppose the singular value decomposition (SVD) of the cofactor matrix $\mathbf Q$ of Eq. (1) is

3 $$\begin{aligned} \mathbf Q = \mathbf U \varvec{\varSigma }\mathbf U', \end{aligned}$$

where $\mathbf U$ is the orthonormal matrix of singular vectors, $\mathbf U'$ is the transpose of $\mathbf U$, and $\varvec{\varSigma }$ is the diagonal matrix of singular values. We assume that the singular values are ordered as $\sigma _1 \ge \sigma _2 \ge \cdots \ge \sigma _n$, where *n* is the number of columns and rows of the matrix $\mathbf Q$. Here, we use Tikhonov regularisation with a unit regularisation matrix. It is equivalent to an approximation $\mathbf Q_{\hbox { tikh}}$ of $\mathbf Q$, which is defined as

4 $$\begin{aligned} \mathbf Q_{\hbox { tikh}} := \mathbf U \varvec{\varSigma }_{\hbox { tikh}} \mathbf U', \end{aligned}$$

where

5 $$\begin{aligned} \varvec{\varSigma }_{\hbox { tikh}} = \varvec{\varSigma }+ \lambda '\,\mathbf I , \end{aligned}$$

and $\mathbf I$ is the unit matrix, and $\lambda '$ is the regularisation parameter. Hence, Tikhonov regularisation with unit regularisation matrix is equivalent to replacing each of the *n* singular values $\sigma _i$ of $\mathbf Q$ with $\sigma _i + \lambda '$. Then, the spectral norm condition number of $\mathbf Q_{\hbox { tikh}}$ reduces from ${\sigma _1 \over \sigma _n}$ to ${\sigma _1 \over \lambda '}$, which when $\lambda ' \gg \sigma _n$ is much smaller than ${\sigma _1 \over \sigma _n}$. The inverse of the regularised cofactor matrix $\mathbf Q_{\hbox { tikh}}$, is then computed as the Caley inverse of $\mathbf Q_{\hbox { tikh}}$, i.e.

6 $$\begin{aligned} \mathbf Q_{\hbox { tikh}}^{-1} = \mathbf U \varvec{\varSigma }_{\hbox { tikh}}^{-1}\,\mathbf U'. \end{aligned}$$

The weighted least-squares estimator of $\mathbf c$,

7 $$\begin{aligned} \hat{\mathbf c}_{\hbox { tikh}} = (\mathbf A' \mathbf Q_{\hbox { tikh}}^{-1} \mathbf A)^{-1} \mathbf A' \mathbf Q_{\hbox { tikh}}^{-1}\,\mathbf d, \end{aligned}$$

is still unbiased, but its dispersion matrix,

8 $$\begin{aligned} D(\hat{\mathbf c}_{\hbox { tikh}}) = \sigma ^2 (\mathbf A' \mathbf Q_{\hbox { tikh}}^{-1} \mathbf A)^{-1}, \end{aligned}$$

is not minimum anymore. In the numerical experiments of Sect. 5, the matrix $\mathbf A' \mathbf Q_{\hbox { tikh}}^{-1} \mathbf A$ appears to be ill-conditioned and requires some regularisation. Here, we use Tikhonov regularisation with unit regularisation matrix and replace the unbiased estimator, Eq. (7), with the biased estimator

9 $$\begin{aligned} \hat{\mathbf c}_{\hbox { tikh,reg}} = (\mathbf A' \mathbf Q_{\hbox { tikh}}^{-1} \mathbf A + \lambda \mathbf I)^{-1} \mathbf A' \mathbf Q_{\hbox { tikh}}^{-1}\,\mathbf d, \end{aligned}$$

where $\lambda$ is another regularisation parameter. In this study, Eq. (9) is referred to as the “regularised weighted least-squares (WLS-reg) estimator”, where “regularised” refers to the regularisation of the cofactor matrix and not to the term $\lambda \mathbf I$ of Eq. (9)

When using Tikhonov regularisation directly applied to the cofactor matrix, we need to find a suitable value of the regularisation parameter $\lambda '$. This can be done, e.g. using a measure of closeness of $\mathbf Q\,\mathbf Q_{\hbox { tikh}}^{-1}$ and $\mathbf Q_{\hbox { tikh}}^{-1} \mathbf Q$, respectively, to the unit matrix $\mathbf I$. As $\mathbf Q$ and $\mathbf Q_{\hbox { tikh}}^{-1}$ do not commute, we may use the symmetric part of this product, i.e.

10 $$\begin{aligned} \tilde{\mathbf I}:= {1 \over 2}(\mathbf Q\,\mathbf Q_{\hbox { tikh}}^{-1} + \mathbf Q_{\hbox { tikh}}^{-1}\,\mathbf Q), \end{aligned}$$

and measure its distance to the unit matrix $\mathbf I$ using a suitable matrix norm, for an overview of matrix norms. However, numerical experiments revealed that the matrix $\tilde{\mathbf I}$ can be indefinite. Then, several popular metrics like the Förstner–Moonen metric (Förstner and Moonen 1999) or the trace of the matrix $\tilde{\mathbf I}$ cannot be used to find a suitable regularisation parameter. The same numerical experiments showed that the log-Euclidean metric and the spectral norm of $\mathbf I - \tilde{\mathbf I}$ decreased monotonously with decreasing regularisation parameter, and therefore, are also not suited to choose the regularisation parameter. Based on these results, we did not use matrix norms to find a suitable regularisation parameter for the noise cofactor matrix, but used the criteria to be discussed in Sect. 5. The same criteria were also used to choose the regularisation parameter of Eq. (9).

### Inversion-free weighted least-squares estimator

According to Grafarend and Schaffrin (1993), there is an equivalent expression for the weighted least-squares estimator, $\hat{\mathbf c} = (\mathbf A' \mathbf Q^{-1} \mathbf A)^{-1} \mathbf A' \mathbf Q^{-1}\,\mathbf d$, which does not require the computation of the inverse of the cofactor matrix:

11 $$\begin{aligned} \hat{\mathbf c} = \mathbf A'(\mathbf A \mathbf A' + \mathbf Q \mathbf B \mathbf Q)^{-1}\,\mathbf d, \end{aligned}$$

where

12 $$\begin{aligned} \mathbf B = \mathbf I - \mathbf A (\mathbf A' \mathbf A)^{-1}\mathbf A'. \end{aligned}$$

In the numerical experiments of Sect. 5, the matrix $\mathbf A' \mathbf A$ in Eq. (12) and the matrix $\mathbf A \mathbf A' + \mathbf Q \mathbf B \mathbf Q$ in Eq. (11) appeared to be ill-conditioned and required some regularisation. Here, we use in both cases Tikhonov regularisation with a unit regularisation matrix. That is, Eq. (11) is replaced by

13 $$\begin{aligned} \hat{\mathbf c}_{\hbox { reg}} = \mathbf A'(\mathbf A \mathbf A' + \mathbf Q \mathbf B_{\hbox { reg}} \mathbf Q + \lambda \mathbf I)^{-1}\,\mathbf d, \end{aligned}$$

with

14 $$\begin{aligned} \mathbf B_{\hbox { reg}} = \mathbf I - \mathbf A (\mathbf A' \mathbf A + {\lambda '} {\mathbf I})^{-1}\mathbf A'. \end{aligned}$$

The two regularisation parameters $\lambda$ and $\lambda '$ are chosen as

15 $$\begin{aligned} \lambda = \lambda _{\hbox { eff}}\, {{{\mathrm{Tr}}}(\mathbf A \mathbf A' + \mathbf Q \mathbf B \mathbf Q) \over n},\;\; \lambda ' = \lambda _{\hbox { eff}}\, {{{\mathrm{Tr}}}(\mathbf A' \mathbf A) \over m}, \end{aligned}$$

where *n* is the number of observations, *m* is the number of parameters, and $\lambda {\hbox { eff}}$ is referred to as the “effective regularisation parameter”. This choice of $\lambda$ and $\lambda '$ implies that the amount of regularisation applied to $\mathbf A' \mathbf A$ when computing $\mathbf B_{\hbox { reg}}$ and applied to $\mathbf A \mathbf A' + \mathbf Q \mathbf B_{\hbox { reg}} \mathbf Q$ when solving the normal equations, respectively, is the same.

### Least-squares solution according to Rao’s unified theory of least-squares

Another approach to deal with an ill-conditioned noise covariance matrix is offered by Rao’s unified theory of least-squares (Rao 1971, 1973, 1978). Though this theory has been developed to address among others rank-deficient noise covariance matrices, we applied it to the ill-conditioned height anomaly noise covariance matrix $\mathbf C$. According to Rao (1971, 1973, 1978), the unbiased, minimum-dispersion estimator for the model of Eq. (1) is

16 $$\begin{aligned} \hat{\mathbf c}_{\hbox { rao}} := (\mathbf A' \mathbf T^-\,\mathbf A)^{-1} \mathbf A' \mathbf T^-\,\mathbf d, \end{aligned}$$

where

17 $$\begin{aligned} \mathbf T = \mathbf Q+\alpha \,\mathbf A \mathbf A', \end{aligned}$$

$\alpha$ is a positive constant, and $\mathbf T^-$ is any symmetric g-inverse of $\mathbf T$. We did some numerical experiments with the setup of Sect. 5 and found that choosing a value of $\alpha$ different from 1 has a negligible effect on the generalised least-squares estimate. In particular, we found that the spectral norm condition number of $\mathbf T$ does not improve when choosing $\alpha$ different from 1. Therefore, the generalised least-squares estimate of Sect. 6 is computed with $\alpha = 1$.

In the numerical experiments of Sect. 6, the normal matrix $\mathbf A' \mathbf T^-\,\mathbf A$ appeared to be ill-conditioned. As in Sects. 4.1 and 4.2, we again used Tikhonov regularisation with a unit regularisation matrix:

18 $$\begin{aligned} \hat{\mathbf c}_{\hbox { rao,reg}} = (\mathbf A' \mathbf T^-\,\mathbf A + \lambda \mathbf I)^{-1} \mathbf A' \mathbf T^-\,\mathbf d. \end{aligned}$$

In this study, we refer to $\hat{\mathbf c}_{\hbox { rao,reg}}$ of Eq. (18) as the “generalised least-squares (GLS) estimator".

Note that the g-inverse $\mathbf T^-$ does not need to be a g-inverse of $\mathbf Q$ (Rao 1978). In the strictly rank-deficient case, it can be computed as the pseudo-inverse of a truncated singular value decomposition of $\mathbf T$, where the truncation index *r* is identical to the rank of $\mathbf T$ where $r < n$. In our case, the choice of the truncation index is not straightforward as the singular value spectrum of $\mathbf T$ gradually decreases to zero without any noticeable gap.

In this study, we test a whole range of truncation indices *q*, and compute the g-inverse $\mathbf T^-$ as

19 $$\begin{aligned} \mathbf T^- = \mathbf U_r \varvec{\varSigma }_r^{-1} {\mathbf U_r}', \end{aligned}$$

where

20 $$\begin{aligned} r = \mathop {{{\mathrm{arg\,min}}}}\limits _q { \Vert \mathbf T \mathbf T_q^- \mathbf T - \mathbf T \Vert \over \Vert \mathbf T \Vert } =: \mathop {{{\mathrm{arg\,min}}}}\limits _q \kappa (q), \end{aligned}$$

and

21 $$\begin{aligned} \mathbf T_q^- = \mathbf U_q \varvec{\varSigma }_q^{-1} \mathbf U_q. \end{aligned}$$

Here, $\varvec{\varSigma }_q$ is the matrix of the *q* largest singular values of $\mathbf T$, and $\mathbf U_q$ is the associated matrix of singular vectors.

### Dispersion matrices

The estimators of Sects. 4.1, 4.2, and 4.3 can be written as

22 $$\begin{aligned} \hat{\mathbf c} = \mathbf S\,\mathbf d, \end{aligned}$$

where the matrix $\mathbf S$ is equal to $(\mathbf A' \mathbf Q_{\hbox { tikh}}^{-1} \mathbf A + \lambda \mathbf I)^{-1} \mathbf A' \mathbf Q_{\hbox { tikh}}^{-1}$, $\mathbf A'(\mathbf A \mathbf A' + \mathbf Q \mathbf B_{\hbox { reg}} \mathbf Q + \lambda \mathbf I)^{-1}$, and $(\mathbf A' \mathbf T^-\,\mathbf A + \lambda \mathbf I)^{-1} \mathbf A' \mathbf T^-$ for the WLS-reg, WLS and GLS estimators, respectively. Then, the dispersion matrix of the estimated SRBF coefficients follows from the law of covariance propagation, i.e.

23 $$\begin{aligned} D(\hat{\mathbf c}) = \sigma ^2( \mathbf S \mathbf Q \mathbf S'), \end{aligned}$$

where $\sigma ^2 \mathbf Q$ is the noise covariance matrix of the data vector $\mathbf d$. Moreover, the dispersion matrix of a linear function $\mathbf A_s\,\hat{\mathbf c}$ of the estimated SRBF coefficients is

24 $$\begin{aligned} D(\mathbf A_s\,\hat{\mathbf c}) = \sigma ^2 \mathbf A_s \mathbf S \mathbf Q \mathbf S' \mathbf A_s^\prime . \end{aligned}$$

## Experimental setup, quality assessment, and parameter choice rule

### Experimental setup

The performance of the afore-mentioned methods were investigated using numerical experiments. We used the experimental setup of Sect. 3. The height anomalies were synthesised on a Reuter grid with $N=240$, comprising 617 data points over the data area. The condition number of the noise covariance matrix $\mathbf C$ is $1.4 \cdot 10^{16}$ (cf. Sect. 3). Contrary to the data used in Sect. 3, we added zero-mean Gaussian noise to the noise-free height anomalies. The noise was generated using a SVD of the height anomaly noise covariance matrix $\mathbf C$. The noisy height anomalies form the elements of the vector $\mathbf d$.

### Quality assessment

To investigate the quality of the weighted least-squares solutions, we use two different measures.

**Fig. 2:**
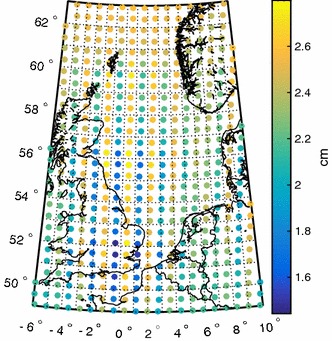
Height anomaly noise standard deviations (in units of cm) of the unregularised GOCO05s GGM complete to degree 200 over the area of interest $49^\circ$–$63^\circ$N and $6^\circ$W–$10^\circ$E

1. From every least-squares estimate $\hat{\mathbf c}$ of the SRBF coefficients, we synthesise height anomalies on a control grid, $\hat{\mathbf d}_s = \mathbf A_s\,\hat{\mathbf c}$. The true height anomalies, $\mathbf d_s$, are synthesised from the spherical harmonic model of the disturbing potential. As a quality measure of a least-squares estimate $\hat{\mathbf c}$, we use the RMS of the errors $\{ \hat{d}_{s,i}-d_{s,i}: i=1 \ldots q\}$, i.e.
  25 $$\begin{aligned} \varepsilon _{\hbox { RMS} }:= { \Vert \hat{\mathbf d}_s - \mathbf d_s\Vert \over \sqrt{q} }. \end{aligned}$$
  For a good least-squares estimate $\hat{\mathbf c}$, $\varepsilon _{\hbox { RMS}}$ should not differ much from the noise SDs of the height anomalies at the control points, which are computed by covariance propagation from the full noise covariance matrix of spherical harmonic coefficients of the unregularised GOCO05s model complete to degree 200. Figure 2 shows a spatial rendition of the noise variances over the area of interest. The noise standard deviations range from 1.6  to 2.7 cm (cf. Sect. 3) and have a mean of $2.3 \pm 0.2$ cm.
2. We compute the dispersion $D(\hat{\mathbf d}_s)$ and compare it with the dispersion $D(\mathbf d_s)$. The latter is computed by covariance propagation from the full noise covariance matrix of spherical harmonic coefficients of the unregularised GOCO05s model. As a quality measure, we use the relative error
  26 $$\begin{aligned} \varepsilon _{\hbox { rel}}:= { \Vert D(\hat{\mathbf d_s}) - D(\mathbf d_s)\Vert \over \Vert D(\mathbf d_s)\Vert }, \end{aligned}$$
  where $\Vert \cdot \Vert$ is the spectral norm.

### Parameter choice rule

Each of the three methods presented in Sect. 4 requires the choice of a suitable regularisation parameter $\lambda$ to stabilise the normal matrix. Moreover, each method requires to fix a second parameter, i.e. $\lambda '$ of Eq. (5) for WLS-reg, $\lambda '$ of Eq. (14) for WLS, and *r* of Eq. (20) for GLS.

When computing the WLS-reg estimate (cf. Sect. 4.1), $\lambda '$ of Eq. (5) is the regularisation parameter of the cofactor matrix. This parameter has an impact on the condition number of the normal matrix of Eq. (9). Therefore, we need to search a two-dimensional parameter space to find suitable values for the two regularisation parameters $\lambda$ and $\lambda '$. The task to choose the two regularisation parameters $\lambda$ and $\lambda '$ when computing the WLS estimate (cf. Sect. 4.2) is reduced to finding the single parameter $\lambda _{\hbox { eff}}$ of Eq. (15). The computation of the GLS estimate (cf. Sect. 4.3) requires to fix $\lambda$ and *r*. For the latter, we use the relative error $\kappa$ of Eq. (20). This makes the search for *r* independent of the search for $\lambda$. Therefore, fixing the two parameters $\lambda$ and *r* reduces to two one-dimensional problems.

Finding suitable values for these parameters requires a parameter choice rule. Standard parameter choice rules such as the *L*-curve (Hansen and O’Leary 1993), generalised cross-validation (Wahba 1990) or variance component estimation (Koch and Kusche 2002) cannot be used here, as there are as many data as SRBF coefficients. In this study, we considered the quality measure $\varepsilon _{\hbox { RMS}}$ of Eq. (25) and $\varepsilon _{\hbox { rel}}$ of Eq. (26) as potential candidates. Numerical experiments revealed that $\varepsilon _{\hbox { rel}}$ is more sensitive to the choice of these parameters than $\varepsilon _{\hbox { RMS}}$. Therefore, we used the minimum of $\varepsilon _{\hbox { rel}}$ as the parameter choice rule. In some cases, this choice also provided the smallest value of $\varepsilon _{\hbox { RMS}}$. If not, we found that the parameter(s), which minimised $\varepsilon _{\hbox { rel}}$ provided a value of $\varepsilon _{\hbox { RMS}}$, which did not differ more than 0.05 mm from the smallest $\varepsilon _{\hbox { RMS}}$.

## Results and discussion

Table 3 shows the main statistics for the three estimators considered in this study. They are complemented by a weighted least-squares estimator, which uses the inverse of the diagonal approximation of the full data noise covariance matrix as weight matrix. In all cases, we only show the statistics for the best solutions, i.e. the ones which minimise $\varepsilon _{\hbox { rel}}$ of Eq. (26).

**Table 3:** The WLS-reg, WLS, and GLS estimates, which minimise $\varepsilon _{\hbox { rel}}$, Eq. (25)

Estimator	$\varepsilon _{\hbox { RMS}}$ (cm)	$\varepsilon _{\hbox { rel}}$	$e_{\hbox { RMS}}$ (cm)	$\lambda _{\hbox { eff}}$	*RD*
WLS-reg, $\lambda '_{\hbox { eff}} = 1.9 \cdot 10^{-2}$	2.71	$1.0 \cdot 10^{-3}$	0.013	$1.0 \cdot 10^{-10}$	0.852
WLS	2.72	$9.8 \cdot 10^{-4}$	0.010	$1.6 \cdot 10^{-12}$	0.866
GLS, $r=492$	2.72	$1.2 \cdot 10^{-3}$	0.082	$1.0 \cdot 10^{-9}$	0.797
WLS-diag	2.72	$9.5 \cdot 10^{-1}$	0.012	$1.0 \cdot 10^{-11}$	0.853

$\lambda '_{\hbox { eff}}$ is the effective regularisation parameter for the data noise covariance matrix; *r* is the truncation index when computing the g-inverse $\mathbf T^-$ using a truncated singular value decomposition; $e_{\hbox { RMS}}$ is the RMS of the least-squares residuals; $\lambda _{\hbox { eff}}$ is the effective regularisation parameter for the regularisation of the normal matrix; *RD* is the model resolution degree of Eq. (27). WLS-diag refers to a weighted least-squares solution, which ignores all covariances of the data noise covariance matrix

Table 3 reveals that the amount of regularisation to be applied to the normal matrix is significantly different among the three estimates. One may expect that the WLS estimate requires more regularisation than the WLS-reg estimate due to the ill-conditioned noise covariance matrix. This is, however, not the case. The effective regularisation parameter is the smallest for the WLS estimate and the largest for the GLS estimate; the latter is a factor 330 larger than the former. This is also reflected in the model resolution degree (*RD*), which is defined as

27 $$\begin{aligned} RD = {{{\mathrm{Tr}}}R \over n},\end{aligned}$$

where *R* is the resolution matrix (i.e. the matrix $\mathbf S \mathbf A$, if the least-squares estimate is $\hat{\mathbf c} = \mathbf S\,\mathbf d$), and *n* is the number of parameters (e.g. Aster et al. 2013). *RD* is an indication of the contribution of the data to the estimated SRBF coefficients in the presence of regularisation; the larger *R*, the higher the contribution of the data to the estimated SRBF coefficients. According to Table 3, WLS-reg and WLS perform the same with a model resolution degree of 85–87%, whereas the model resolution degree for GLS is smaller, about 80%. The somehow lower model resolution degree for GLS may be due to the choice of the g-inverse of the matrix $\mathbf T$ of Eq. (17), which is a challenging task due to the gradually decreasing singular value spectrum of this matrix.

**Fig. 3:**
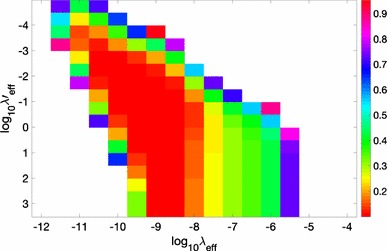
Parameter choice for the regularised weighted least-squares estimator (WLS-reg). $\varepsilon _{\hbox { rel}}$ in percentage as function of $\lambda '_{\hbox { eff}}$ and $\lambda _{\hbox { eff}}$. Values larger than 1% are shown in white for better readability. The minimum of $\varepsilon _{\hbox { rel}} = 1.0 \cdot 10^{-3} = 0.1\%$ is attained for $\lambda '_{\hbox { eff}} = 1.9 \cdot 10^{-2}$ and $\lambda _{\hbox { eff}} = 1 \cdot 10^{-10}$

**Fig. 4:**
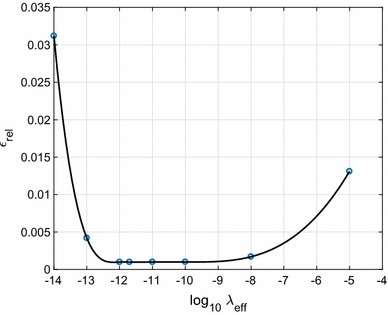
Parameter choice for the weighted least-squares estimator. $\varepsilon _{\hbox { rel}}$ as function of $\lambda _{\hbox { eff}}$. The minimum is attained at $\lambda _{\hbox { eff}} = 1.6 \cdot 10^{-12}$

**Fig. 5:**
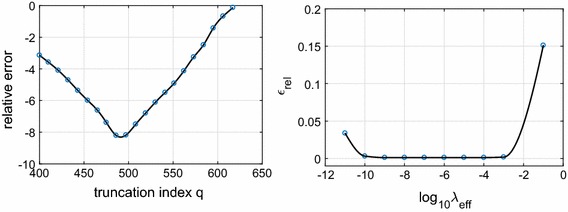
Parameter choice for the generalised least-squares estimator. Left: relative error $\kappa$ as function of the truncation index *q*. The minimum is attained at $q=r=492$. The relative error is $\kappa (492) = 3.2 \cdot 10^{-9}$. Right: $\varepsilon _{\hbox { rel}}$ as function of $\lambda _{\hbox { eff}}$ for $q = r = 492$. The minimum is attained at $\lambda _{\hbox { eff}} = 1 \cdot 10^{-9}$

Figure 3 shows $\varepsilon _{\hbox { rel}}$ as function of $(\lambda _{\hbox { eff}}, \lambda '_{\hbox { eff}})$. The minimum is attained at $(\lambda '_{\hbox { eff}} = 1.9 \cdot 10^{-2},\lambda _{\hbox { eff}} = 1.0 \cdot 10^{-10})$. A value of $\lambda _{\hbox { eff}} = 1.0 \cdot 10^{-10}$ indicates that the WLS-reg estimate is more sensitive to the regularisation of the normal matrix compared to the WLS estimate (cf. Fig 4) and the GLS estimate (cf. Fig 5), respectively. Figure 3 also shows that a good least-squares solution requires a heavy regularisation of the noise covariance matrix. The solution which minimises $\varepsilon _{\hbox { rel}}$ is obtained with an effective regularisation parameter $\lambda '_{\hbox { eff}} = 1.9 \cdot 10^{-2}$. Solutions not that far from the optimal one are also obtained for effective regularisation parameters $\lambda '_{\hbox { eff}}$ of the order of O(1) or larger. Such an exceptionally strong regularisation is unexpected in the sense that it is orders of magnitude stronger than one would expect based on the singular value spectrum of the noise covariance matrix and the effect of rounding errors on the computed inverse.

Figure 4 shows $\varepsilon _{\hbox { rel}}$ as function of $\lambda _{\hbox { eff}}$ for the WLS estimate. The minimum is attained at $\lambda _{\hbox { eff}} = 1.6 \cdot 10^{-12}$. However, the curve is very flat over a broad range of effective regularisation parameters between about $10^{-12}$ and $10^{-8}$. The corresponding least-squares estimates of the SRBF coefficients are very close to each other, and the estimates do not differ more than 0.02 cm in terms of height anomalies over the area of interest. We consider this as a positive result as it makes it easy to find a suitable regularisation parameter.

The results for the GLS estimate are shown in Fig 5. The left plot of Fig 5 shows the relative error $\kappa$ of Eq. (20) as function of the truncation index *q*. A clear minimum is attained at $q = r = 492$. The relative error is $\kappa (492) = 3.2 \cdot 10^{-9}$. This indicates that finding a good g-inverse $\mathbf T^-$ using Eq. (19) may be possible with a truncated singular value decomposition despite the gradually decreasing singular value spectrum of $\mathbf T$. Whether this applies to other datasets and areas of interest, as well, remains open, and may be considered as a weak point of the GLS estimator. The right plot of Fig. 5 shows $\varepsilon _{\hbox { rel}}$ as function of the effective regularisation parameter $\lambda _{\hbox { eff}}$, which is used to regularised the normal matrix. The minimum is attained at $\lambda _{\hbox { eff}} = 1 \cdot 10^{-9}$. Similar to what was found for the WLS estimate, the curve is very flat around the minimum over a broad range of effective regularisation parameters from $10^{-10}$ to $10^{-3}$. Again, the corresponding least-squares estimates of the SRBF coefficients are almost identical. In terms of height anomalies, the solutions do not differ more than 1 mm.

Table  3 shows that the smallest value of $\varepsilon _{\hbox { rel}}$ is about $10^{-3}$ for the three methods investigated in this paper. Hence, the noise covariance matrix of the height anomalies at the control grid always agrees very well with the noise covariance matrix directly propagated from the noise covariance matrix of the spherical harmonic model of the disturbing potential. The same applies to the fit of each solution to the control data; the RMS misfit is $\varepsilon _{\hbox { RMS}} = 2.7$ cm for all three methods. This is at the upper limit of the height anomaly noise standard deviations directly propagated from the noise covariance matrix of GOCO05s over the area of interest (which range from 1.6  to 2.7 cm, cf. Fig 2). The estimator that ignores the data noise covariances provides the same RMS misfit of 2.7 cm. However, the error $\varepsilon _{ \text{ rel }}$ is 0.95, i.e. almost three orders of magnitude larger than for the other three estimators. This is in line with what we expect from theory. When applying weighted least-squares to a single dataset, errors in the data noise covariance matrix have a minor effect on the least-squares estimate, but a significant effect on the dispersion of linear functionals of the estimate.

**Fig. 6:**
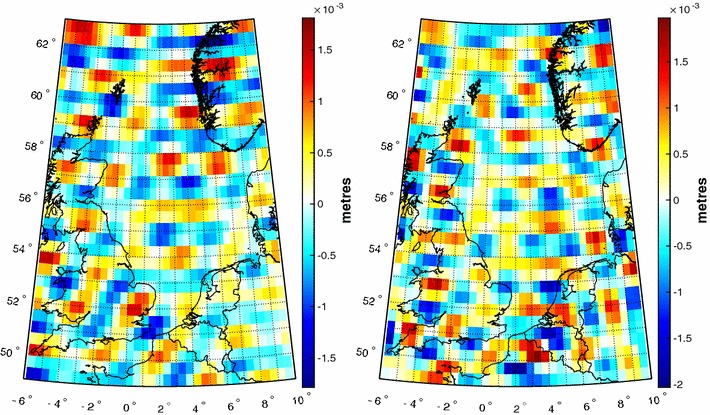
Spatial rendition of the differences WLS estimate minus WLS-reg estimate (left) and WLS estimate minus GLS estimate (right). The maximum absolute difference is 0.3 mm (left) and 2.3 mm (right)

Figure 6 shows a spatial rendition of the differences WLS estimate minus WLS-reg estimate and WLS estimate minus GLS estimate, respectively, in terms of height anomalies at the control points. The spatial patterns are random, indicating that there are no systematic differences between the three estimates. The differences are very small; the maximum absolute difference between the WLS estimate on the one hand and the WLS-reg estimate and the GLS estimate on the other hand is just 0.3  and 2.3 mm, respectively.

**Fig. 7:**
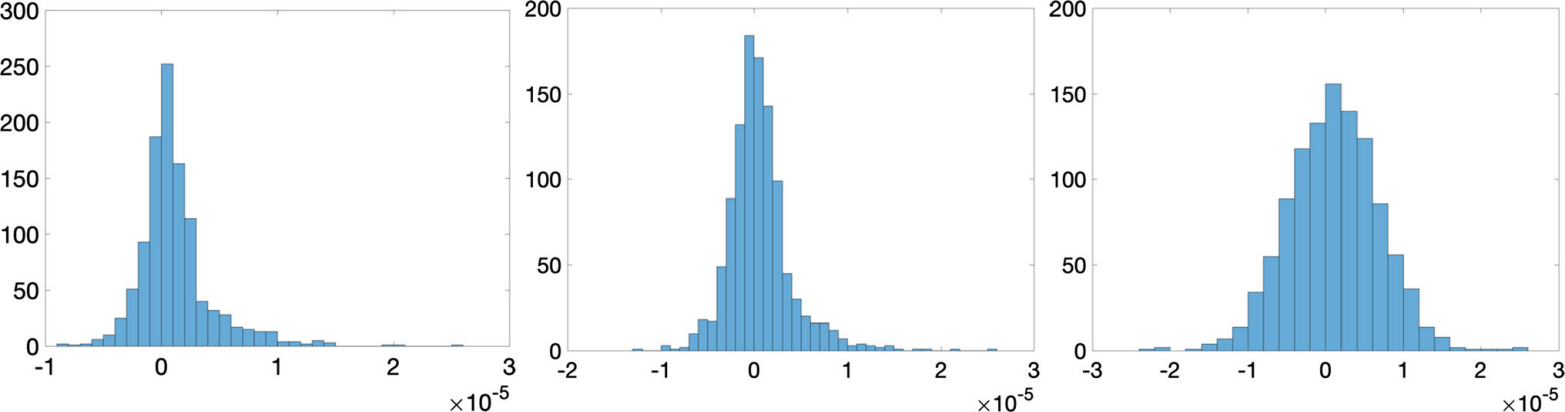
Histogram of differences in height anomaly noise standard deviations at the control points in units of metres. From left to right: WLS-reg estimate, WLS estimate, and GLS estimate. The differences are comparable for the WLS-reg estimate and the WLS estimate, but larger for the GLS estimate

$\varepsilon _{\hbox { rel}}$ measures the difference in the spectral norm between the height anomaly noise covariance matrix associated with a least-squares estimate and the one directly propagated from the noise covariance matrix of spherical harmonic coefficients of the disturbing potential. A measure which is easier to interpret are the differences in the standard deviations of the two noise covariance matrices at the control data points as shown in Fig 7. The differences are comparable for the WLS-reg estimate and WLS estimate and significantly larger for the GLS estimate.

## Summary and conclusions

In this study, we investigated three methods to deal with the high condition number of the noise covariance matrix of a state-of-the-art GGM after propagation into gravity field functionals over a local data area. This problem is relevant when estimating a local model of the disturbing potential considering all available datasets as being noisy.

We showed that the noise covariance matrix of height anomalies propagated from the full noise covariance matrix of GOCO05s, has a gradually decreasing singular value spectrum without any noticeable gap and a high condition number. The latter depends among others on the density of the points where the height anomalies are synthesised. The density has to be chosen high enough to guarantee that the error of the local SRBF model of the disturbing potential is negligible compared to the effect of the noise in the data. We showed that this requires a point density, which is higher than the maximum degree of the GGM suggests. The associated height anomaly noise covariance matrix had a condition number larger than the reciprocal value of the relative rounding error unit of IEEE 754 double precision arithmetic. Therefore, a straightforward computation of the weight matrix would imply the loss of all significant digits.

To deal with the high condition number of the noise covariance matrix, we investigated three methods: Tikhonov regularisation of the noise covariance matrix in combination with the standard formula of the weighted least-squares estimator, an alternative formula of the weighted least-squares estimator, which does not require to compute the inverse of the noise covariance matrix, and Rao’s generalised least-squares estimator. Our experiments indicate that these methods and the main findings of Sect. 6 are not dependent on the chosen experimental setup.

**Table 4:** SRBF model error in terms of height anomalies (in units of cm) as a function of the extension of the parameterisation area beyond the data area

Extension	# SRBFs	# Data points	Min (cm)	Max (cm)	Mean (cm)	SD (cm)
$0^\circ$	435	435	$-\,6.05$	5.85	0.05	2.20
$1^\circ$	503	435	$-\,6.44$	6.07	0.05	2.07
$2^\circ$	576	435	$-\,6.51$	5.70	0.03	2.29
$3^\circ$	650	435	$-\,6.35$	6.96	0.01	2.36
$4^\circ$	761	435	$-\,6.51$	6.32	0.00	2.34
$5^\circ$	850	435	$-\,6.66$	6.06	0.01	2.32

The Poisson wavelet is used. Data are not oversampled. If the number of SRBFs exceeds to number of data, a minimum norm solution is computed

We showed that all three methods provide least-squares estimates of the SRBF coefficients which were identical within noise. Moreover, the dispersion matrices of the estimated SRBF coefficients and of height anomalies at a set of control points agreed very well with each other and with the height anomaly noise covariance matrix directly propagated form the full noise covariance matrix of spherical harmonic coefficients of the GGM. Prerequisite was that the two regularisation parameters each of the three methods knows, were chosen properly. We demonstrated that a parameter choice rule which uses the dispersion matrix of height anomalies at a control dataset allowed such a choice.

Among the three methods, we prefer the inversion-free weighted least-squares estimator. We showed that the choice of the two regularisation parameters can be reduced successfully to the choice of a single parameter, and the least-squares estimate and its dispersion matrix appeared to be quite robust against this choice in the numerical experiments. The fact that the inversion-free weighted least-squares estimator requires the solution of a system of linear equations of a size equal to the number of data does not pose numerical problems in real applications. We found that the weak point of Rao’s generalised least-squares estimator is the need to compute a g-inverse of a matrix with a gradually decreasing singular value spectrum without any noticeable gap. In this study, we computed such a g-inverse successfully with a truncated singular value decomposition. However, whether this applies to other situations than considered in this study, is an open question. Using Tikhonov regularisation of the data noise covariance matrix in combination with the standard formula for the weighted least-squares estimator, required an exceptionally strong regularisation of the data noise covariance matrix to obtain a good least-squares estimate of the SRBF coefficients and an accurate dispersion matrix of the estimated SRBF coefficients. We found that the major drawback of this method is the need to search a two-dimensional space to find optimal values of the two regularisation parameters.

## Appendix A: Extension of the parameterisation area

In this appendix, we investigate whether an extension of the parameterisation area beyond the data area provides a model error, which does not require any oversampling. Such an extension is suggested in several papers about local gravity field modelling using SRBFs (e.g. Naeimi 2013; Bentel et al. 2013a; Eicker et al. 2014; Bucha et al. 2016). In the following, the setup chosen in the numerical experiments of Sect. 5 (i.e. Poisson wavelets and data points on a Reuter grid with control parameter 240, denoted RG 240, no extension of the parameterisation area) is referred to as the “reference setup”. Moreover, the area of interest and the data area are the same as in the reference setup.

The disturbing potential used in the numerical experiments of Sect. 5 is limited to a maximum degree 200. Therefore, we located the poles of the Poisson wavelets on RG 201, and generated height anomalies on RG 201 (i.e. no oversampling).

Table 4 shows the model error statistics for various extensions of the parameterisation area ranging from $0^\circ$ to $5^\circ$.

It appears that an extension of the parameterisation area without oversampling does not improve the model error. In particular, the maximum absolute model error is about 6 cm. This is even larger than the effect of data noise on the estimated quasi-geoid model, which was found to have a standard deviation between 1.6  and 2.7 cm over the area of interest.

**Table 5:** SRBF model error in terms of height anomalies (in units of cm) as a function of the extension of the parameterisation area beyond the data area

Extension	# SRBFs	# Data points	Min (cm)	Max (cm)	Mean (cm)	SD (cm)
$0^\circ$	435	516	$-\,5.36$	4.70	0.12	1.62
$1^\circ$	503	516	$-\,0.95$	1.33	0.01	0.28
$2^\circ$	576	516	$-\,1.46$	1.06	0.00	0.32
$3^\circ$	650	516	$-\,0.85$	0.77	0.01	0.24
$4^\circ$	761	516	$-\,0.60$	0.74	0.00	0.19
$5^\circ$	850	516	$-\,0.76$	0.80	0.00	0.20

The Poisson wavelet is used. Data are oversampled by a factor of 1.1. If the number of SRBFs exceeds to number of data, a minimum norm solution is computed

**Table 6:** SRBF model error in terms of height anomalies (in units of cm) as a function of the extension of the parameterisation area beyond the data area

Extension	# SRBFs	# Data points	Min (cm)	Max (cm)	Mean (cm)	SD (cm)
$0^\circ$	435	617	$-\,5.44$	5.54	0.25	1.74
$1^\circ$	503	617	$-\,0.87$	0.94	-$\,0.01$	0.37
$2^\circ$	576	617	$-\,0.45$	0.39	0.00	0.08
$3^\circ$	650	617	$-\,0.24$	0.20	0.00	0.05
$4^\circ$	761	617	$-\,0.37$	0.28	0.00	0.07
$5^\circ$	850	617	$-\,0.16$	0.13	0.00	0.03

The Poisson wavelet is used. Data are oversampled by a factor of 1.2. If the number of SRBFs exceeds to number of data, a minimum norm solution is computed

**Table 7:** SRBF model error in terms of height anomalies (in units of cm) as a function of the extension of the parameterisation area beyond the data area

Extension	# SRBFs	# Data points	Min (cm)	Max (cm)	Mean (cm)	SD (cm)
$0^\circ$	435	617	$-\,4.95$	4.60	0.00	1.77
$1^\circ$	503	617	$-\,1.03$	1.06	0.00	0.42
$2^\circ$	576	617	$-\,0.43$	0.40	0.00	0.07
$3^\circ$	650	617	$-\,0.28$	0.25	0.00	0.07
$4^\circ$	761	617	$-\,0.42$	0.42	0.00	0.09
$5^\circ$	850	617	$-\,0.08$	0.07	0.00	0.02

The Shannon kernel is used. Data are oversampled by a factor of 1.2. If the number of SRBFs exceeds to number of data, a minimum norm solution is computed

In the second experiment, we wanted to investigate whether an extension of the parameterisation area in combination with some oversampling (which should be less than the oversampling factor of 1.2, we used in the reference setup), provides a smaller model error. The setup was identical to the one in the previous experiment except that the data were located on RG 221, which corresponds to an oversampling factor of 1.1. From the results shown in Table 5, we conclude that (i) an extension of the parameterisation area beyond the data area in combination with an oversampling factor of 1.1 indeed reduces the model error; (ii) though the improvement is significant, it is still not sufficient. That is, the smallest maximum absolute model error of 0.7 cm is attained for a $4^\circ$ extension, which is still a factor of 3.5 larger than for the reference setup. The condition number of the data noise covariance matrix is $6.2 \cdot 10^{12}$. Computing a weighted least-squares estimate of the quasi-geoid model without applying any regularisation to this data noise covariance matrix provided a useless solution, indicating that some regularisation is needed.

In the next experiment, we located the poles of the Poisson wavelets on RG 201 and the data on RG 240. This corresponds to the same oversampling factor of 1.2 as used in the reference setup. Table 6 shows that now, an extension of the parameterisation area provides indeed a reduction of the maximum absolute model error to a level comparable to or even below the values obtained for the reference setup. For instance, an extension of $3^\circ$ gives a maximum absolute model error of 0.2 cm, which is identical to the one we found for the reference setup.

Finally, we repeated the last experiment now using the Shannon kernel instead of the Poisson wavelet. The SRBF poles were located on RG 201, and the data points were located on RG 240 (i.e. the oversampling factor is equal to 1.2). Table 7 shows that the results are almost identical to the results with the Poisson wavelet, which are shown in Table 6.

Based on the numerical results, we conclude that even when we extend the parameterisation area beyond the data area, we need to oversample by at least a factor of 1.2 (i.e. with the same factor as used in the reference setup), to obtain a maximum absolute model error which is a factor of 5–10 smaller than the effect of data noise on the estimated quasi-geoid model. Moreover, the results shown in Tables 6 and 7 demonstrate that the model error is essentially identical no matter whether the Poisson wavelet of the Shannon kernel is used.

## Appendix B: Least-squares data combination in local quasi-geoid modelling

The motivation for this study is local quasi-geoid modelling using a GGM as a (low-resolution) noisy dataset and combining it with (high-resolution) local noisy datasets, e.g. terrestrial gravity anomalies, airborne gravity disturbances and along-track quasi-geoid height differences from satellite radar altimetry, using weighted least-squares.

There are several options for the combination of a low-resolution GGM dataset with high-resolution datasets. For instance, one may complete the low-resolution GGM dataset and its noise covariance matrix to make it spectrally consistent with the high-resolution datasets (e.g. Schuh et al. 2015). Alternatively, one may low-pass filter the high-resolution datasets to make them spectrally consistent with the low-resolution GGM dataset. The latter requires a careful choice of the functional model for the high-resolution and the low-resolution datasets, respectively, which for broadband signals is frequently offered by a multi-scale model (e.g. Chambodut et al. 2005; Lieb et al. 2016). Moreover, as extensively discussed in this paper, the noise covariance matrix of the GGM dataset may be ill-conditioned. The disadvantage of the former approach is that the completion of the low-resolution GGM dataset requires some assumptions about the power spectrum of the Earth’s gravity field, isotropy and stationarity (e.g. Schuh et al. 2015). Though the completed noise covariance matrix is invertible on a global scale, there is no guarantee that this still applies for data synthesised over a local domain in local quasi-geoid modelling. For instance, when we just use the diagonal elements of the noise covariance matrix of GOCO05s in the spherical harmonic domain and propagate it into height anomalies (or gravity anomalies) on a local grid (where the grid size is properly chosen to avoid loss of information, see Sect. 2), the condition number of the propagated noise covariance matrix is as high as when using the full noise covariance matrix in the spherical harmonic domain.

There are several options for the combination of a low-resolution GGM dataset with high-resolution datasets. For instance, one may complete the low-resolution GGM dataset and its noise covariance matrix to make it spectrally consistent with the high-resolution datasets (e.g. Schuh et al. 2015). Alternatively, one may low-pass filter the high-resolution datasets to make them spectrally consistent with the low-resolution GGM dataset. The latter requires a careful choice of the functional model for both the high-resolution and the low-resolution datasets, which for broadband signals is frequently offered by a multi-scale model (e.g. Chambodut et al. 2005; Lieb et al. 2016). Moreover, as extensively discussed in this paper, the noise covariance matrix of the GGM dataset may be ill-conditioned. The disadvantage of the former approach is that the completion of the low-resolution GGM dataset requires some assumptions about the power spectrum of the Earth’s gravity field, isotropy and stationarity (e.g. Schuh et al. 2015). Though the completed noise covariance matrix is invertible on a global scale, there is no guarantee that this still applies for data synthesised over a local domain in local quasi-geoid modelling. For instance, even if the data noise covariance matrix in the spherical harmonic domain is a diagonal matrix, the data noise covariance matrix of any functional synthesised at a local sufficiently dense grid will have a similar condition number as the ones discussed in Sect. 2.

Here, we prefer to use the original low-resolution GGM dataset and noise covariance matrix. The approach of data combination is identical to Klees et al. (2017). Using this approach, we illustrate how the methods investigated in this paper can be exploited when combining the GGM dataset with high-resolution datasets.

The low-resolution GGM dataset $\{d_1(x_{1k}): k = 1 \ldots K_1\}$ is synthesised from the spherical harmonic coefficients of the GGM as

28 $$\begin{aligned} d_1(x_{1k})= & {} \sum _{n=0}^{L_1} \sum _{m=1}^{2n+1} \Big (\hat{c}_{nm} - c_{nm}^{(\tiny {\hbox {ref}})}\Big )\, (F_1\, H_{nm})(x_{1k}), \nonumber \\&\quad k=1 \ldots K_1, \end{aligned}$$

where $\{\hat{c}_{nm}\}$ are the spherical harmonic coefficients of the GGM, $\{c_{nm}^{(\tiny {\hbox {ref}})}\}$ are the spherical harmonic coefficients of the reference GGM, $H_{nm}$ is a solid spherical harmonics of degree *n*, and $F_1$ is the height anomaly functional. The low-resolution dataset is band-limited to a degree $L_1 \le L_{\tiny \hbox {GGM}}$, where $L_{\tiny \hbox {GGM}}$ is the maximum degree of the GGM. The high-resolution datasets are denoted $\{d_2(x_{2k}): k=1 \ldots K_2\}$; we assume that they allow the resolution of wavelengths up to a maximum degree $L_2$. Defining a kernel

29 $$\begin{aligned} \delta _L(x,y)= & {} \sum _{n=0}^L {1 \over 4\pi R^2}\Big ({R \over |x|}\Big )^{n+1}\Big ({R \over |y|}\Big )^{n+1}\,Q_n(\hat{x} \cdot \hat{y}),\nonumber \\&\quad x,\,y \in \overline{\hbox {ext} \sigma _R}, \end{aligned}$$

a spherical convolution of *T* with $\delta _L$ as

30 $$\begin{aligned} (\delta _L * T)(x) = \int _{\sigma _R} \delta _L(x,y) T(y)\,d\sigma _R(y), \end{aligned}$$

and linear functionals $F_{2k}$ of the disturbing potential *T*, we may relate the datasets $d_1$ and $d_2$ to the disturbing potential *T* as

31 $$\begin{aligned} E\{d_1\}(x_{1k})&= F_1 (\delta _{L_1} * T)(x_{1k}), \quad k=1 \ldots K_1, \end{aligned}$$

32 $$\begin{aligned} E\{d_2\}(x_{2k})&= F_{2k} (\delta _{L_2} * T)(x_{2k}),\quad k=1 \ldots K_2, \end{aligned}$$

where $E\{\cdot \}$ denotes mathematical expectation. The local model of *T* is a two-scale model, i.e.

33 $$\begin{aligned} T(x) = \sum _{i=1}^{I_1} c_{1 i}\,\varPsi _1(x,z_{1 i}) + \sum _{i=1}^{I_2} c_{2 i}\,\varPsi _2(x,z_{2 i}). \end{aligned}$$

The first term on the right-hand side of Eq. (33) is a low-resolution model of *T* comprising degrees from 0 to $L_1$, i.e. its resolution is identical to the resolution of dataset $d_1$. The second term on the right-hand side of Eq. (33) complements the low-resolution model to the maximum resolution $L_2$ of dataset $d_2$. In the context of a multi-resolution analysis, it represents a detail space comprising wavelengths from degrees $L_1+1$ to $L_2$. The basis functions $\varPsi _1$ and $\varPsi _2$ of Eq. (33) are defined as

34 $$\begin{aligned} \varPsi _1(x,z)&= (P* \varPhi )(x,z), \end{aligned}$$

35 $$\begin{aligned} \varPsi _2(x,z)&= (\delta _{L_2} - P) * \varPhi (x,z), \end{aligned}$$

where

36 $$\begin{aligned} \varPhi (x,z_i)= & {} {R \over |x|}\,\sum _{l=0}^{L_2}\, \phi _l\,\Big ({|z_i| \over |x|}\Big )^l\,Q_l(\hat{x} \cdot \hat{z}_i),\nonumber \\&\quad x \in \overline{\hbox {ext} \sigma _R}, \; z_i \in \hbox {int} \sigma _R, \end{aligned}$$

is a SRBF with pole at $z_i$, $Q_l$ is the reproducing kernel of the space of harmonic functions of degree *l*, $\phi _l$ is the Legendre coefficient of degree *l*, $\hat{x} = {x \over |x|}$ and $\hat{z}_i = {z_i \over |z_i|}$ are points on the unit sphere, and $\sigma _R$ is the surface of a sphere of radius *R*. The kernel *P* is defined as

37 $$\begin{aligned} P(x,y)= & {} \sum _{n=0}^\infty {1 \over 4\pi R^2}\Big ({R \over |x|}\Big )^{n+1}\Big ({R \over |y|}\Big )^{n+1}\,h_n\, Q_n(\hat{x} \cdot \hat{y}),\nonumber \\&\quad x,\,y \in \overline{\hbox {ext} \sigma _R} .\end{aligned}$$

The Legendre coefficients $\{h_n: n = 0,1,2,\ldots \}$ are equal to 1 for degrees $n \le p_1$, taper off between degrees $p_1< n < p_2$, and are zero for all degrees $n \ge p_2$. An example is a cosine taper,

38 $$\begin{aligned} h_n = {\left\{ \begin{array}{ll} 1, &{} n < p_1 \\ 0.5 + 0.5 \cos \Big ( \pi {n - p_1 \over p_2 - p_1} \Big ), &{} p_1 \le n \le p_2 \le L_2 \\ 0, &{} n > p_2 \end{array}\right. }. \end{aligned}$$

Alternatively, $P * \varPhi$ may be a Blackman scaling function (e.g. Schmidt et al. 2007). The coefficients $\{c_{1i}\}$ and $\{c_{2i}\}$ of Eq. (33) are estimated in two steps. First, we use the functional model

39 $$\begin{aligned} E\{d_2\}(x_{2k})= & {} \sum _{i=1}^{I_2} c_{2i} \,F_{2k} (\delta _{L_2} *\varPhi )(x_{2k},z_i), \nonumber \\&\quad k=1 \ldots K_2, \end{aligned}$$

and estimate the coefficients $\{c_{2i}\}$ using weighted least-squares. Suppose $\{\hat{c}_{2i}\}$ are the least-squares estimates of $\{c_{2i}\}$. Then, we define a new dataset

40 $$\begin{aligned} d_3(x_{1k}) := \sum _{i=1}^{I_1} \hat{c}_{2i}\,(F_1 \varPsi _1)(x_{1k},z_{1i}),\quad k=1 \ldots K_1. \end{aligned}$$

The resolution of the dataset $d_3$ is identical to the resolution of the dataset $P * d_1$. In that sense, $d_3$ and $P*d_1$ are spectrally consistent. Then, we use the functional model

41 $$\begin{aligned}&\begin{pmatrix} (P*E\{d_1\})(x_{1k}) \\ E\{d_3\}(x_{1k}) \end{pmatrix} = \sum _{i=1}^{I_1} c_{1i}\, (F_1 \varPsi _1)(x_{1k},z_{1i}),\nonumber \\&\quad k=1 \ldots K_1 \end{aligned}$$

to estimate the coefficients $\{c_{1i}\}$, using weighted least-squares techniques. The noise covariance matrix of dataset $d_3$ is computed from the noise covariance matrix of the estimated coefficients $\{\hat{c}_{2i}\}$ using the law of covariance propagation. Like the noise covariance matrix of the dataset $\{P*E\{d_1\} \}$, it is a full matrix. If $\{\hat{c}_{1i}\}$ are the least-squares estimates of $\{c_{1i}\}$, the least-squares estimate of the disturbing potential it given as

42 $$\begin{aligned} \hat{T}(x) = \sum _{i=1}^{I_1} \hat{c}_{1 i}\,\varPsi _1(x,z_{1 i}) + \sum _{i=1}^{I_2} \hat{c}_{2 i}\,\varPsi _2(x,z_{2 i}). \end{aligned}$$
